# Post-Traumatic Epilepsy After Mild and Moderate Traumatic Brain Injury: A Narrative Review and Development of a Clinical Decision Tool

**DOI:** 10.3390/reports8040193

**Published:** 2025-09-29

**Authors:** Ioannis Mavroudis, Katerina Franekova, Foivos Petridis, Alin Ciobica, Gabriel Dăscălescu, Carmen Rodica Anton, Ciprian Ilea, Sotirios Papagiannopoulos, Dimitrios Kazis, Emil Anton

**Affiliations:** 1 Department of Neuroscience, Leeds Teaching Hospital, NHS Trust, Leeds LS17 7HY, UK; ioannis.mavroudis@gmail.com; 2 Institute of Health Sciences, University of Leeds, Leeds LS2 9NL, UK; katerina.franekowa@gmail.com; 3 Third Department of Neurology, Aristotle University of Thessaloniki, 54124 Thessaloniki, Greece; f_petridis83@yahoo.gr (F.P.); spapagia@auth.gr (S.P.); dimitrios.kazis@gmail.com (D.K.); 4 Academy of Romanian Scientists, 050094 Bucharest, Romania; 5 Department of Biology, Faculty of Biology, “Alexandru Ioan Cuza” University of Iasi, 700505 Iasi, Romania; alin.ciobica@uaic.ro (A.C.); gabidascalescu2001@gmail.com (G.D.); 6 CENEMED Platform for Interdisciplinary Research, University of Medicine and Pharmacy “Grigore T. Popa”, 700115 Iasi, Romania; 7 “Olga Necrasov” Center, Depart of Biomedical Research, Romanian Academy, 700505 Iasi, Romania; 8 “Ioan Haulica” Institute, Apollonia University, 700511 Iasi, Romania; 9 Faculty of Medicine, “Grigore T. Popa” University of Medicine and Pharmacy, 700115 Iasi, Romania; ilea.ciprian@umfiasi.ro (C.I.); emil.anton@umfiasi.ro (E.A.)

**Keywords:** post-traumatic epilepsy (PTE), mild traumatic brain injury (mTBI), moderate TBI, seizure risk, contusion, early seizures, EEG, neuroimaging, prediction model, clinical decision tool

## Abstract

Background: Post-traumatic epilepsy (PTE) is a recognized complication of traumatic brain injury (TBI), yet its risk following mild and moderate TBI remains underappreciated. Although mild TBI represents the majority of cases in clinical practice, a subset of patients develop unprovoked seizures months or even years post-injury. This review aims to synthesize current evidence on the incidence and predictors of PTE in mild and moderate TBI and to propose a clinically actionable decision-support tool for early risk stratification. Methods: We performed a narrative review of peer-reviewed studies published between 1985 and 2024 that reported on the incidence, risk factors and predictive models of PTE in patients with mild (Glasgow Coma Scale [GCS] 13–15) and moderate (GCS 9–12 or imaging-positive) TBI. Data from 24 studies were extracted, focusing on neuroimaging findings, early post-traumatic seizures, EEG abnormalities and clinical risk factors. These variables were integrated into a rule-based algorithm, which was implemented using Streamlit to enable real-time clinical decision-making. The decision-support tool incorporated five domains: injury severity, early post-traumatic seizures, neuroimaging findings (including contusion location and hematoma type), clinical and demographic variables (age, sex, psychiatric comorbidities, prior TBI, neurosurgical intervention) and EEG abnormalities. Results: PTE incidence following mild TBI ranged from <1% to 10%, with increased risk observed in patients presenting with intracranial hemorrhage or early seizures. From moderate TBI, incidence rates were consistently higher (6–12%). Key predictors included early seizures, frontal or temporal contusions, subdural hematoma, multiple contusions and midline shift. Additional risk-enhancing factors included prolonged loss of consciousness, male sex, psychiatric comorbidities and abnormal EEG patterns. Based on these features, we developed a decision-support tool that stratifies patients into low-, moderate- and high-risk categories for developing PTE. Conclusions: Even in non-severe cases, patients with mild and moderate TBI who exhibit high-risk features remain vulnerable to long-term epileptogenesis. Our proposed tool provides a pragmatic, evidence-based framework for early identification and follow-up planning. Prospective validation studies are needed to confirm its predictive accuracy and optimize its clinical utility.

## 1. Introduction

Traumatic brain injury (TBI) is a major public health concern and remains one of the most prevalent causes of acquired epilepsy worldwide, affecting both civilian and military populations [1]. Among its long-term neurological consequences, post-traumatic epilepsy (PTE) occupies a central place due to its chronicity, impact on quality life and contribution to neurocognitive decline. PTE is defined as the occurrence of recurrent, unprovoked seizures that manifest more than seven days following a traumatic insult to the brain [1,2]. While the link between severe TBI and epileptogenesis is well documented, with reported incidence rates ranging from 30% to 50% depending on lesion location, severity and duration of follow-ups [2,3], the potential for epilepsy following mild (GCS 13–15) and moderate (GCS 9–12) TBI remains insufficiently recognized and often underestimated in clinical practice.

The vast majority of TBIs are classified as mild, particularly in emergency, sports medicine and combat settings. Despite this, a significant subset of these patients later develop delayed-onset neurological sequelae, including PTE, especially when risk-modifying factors are present. These include intracranial hemorrhagic lesions, early post-traumatic seizure and structural abnormalities identifiable through advanced neuroimaging [4]. Moderate TBI, though comparatively less studied in isolation, represents a distinct clinical phenotype with a unique neuropathological trajectory. Patients with moderate TBI often exhibit imaging-positive findings, such as cortical contusions, subdural hematomas or diffuse axonal injury, even in the presence of intermediate GCS scores, suggesting a more complex relationship between injury severity and long-term outcomes [4,5].

Recent studies highlight considerable heterogeneity in the reported incidence of PTE following mild and moderate TBI. For instance, rates range from < 1% in uncomplicated concussions with normal neuroimaging to over 10% in patients exhibiting structural brain lesions, hemorrhages or early seizures [5,6,7]. Notably, even a single mild TBI has been associated with a more than two-fold increase in epilepsy risk compared to the general population [7,8]. Cumulative injury, such as in athletes or military personnel with repetitive head trauma, further amplifies this risk through a dose-response relationship [8]. In moderate TBI, especially those involving temporal and frontal lobe contusions and subdural hematomas, the risk of PTE frequently exceeds 5–10%, particularly when accompanied by early seizures, prolonged loss of consciousness or midline shift [9].

The pathogenesis of PTE is multifactorial, involving excitotoxicity, neuroinflammation, gliosis, blood-brain barrier disruptions and maladaptive synaptic remodeling. These biological cascades are often initiated acutely but may evolve subclinically over weeks to months before culminating in spontaneous seizure activity. Despite this knowledge, few predictive tools exist that can reliably stratify seizure risk in patients with non-severe TBI.

Risk models incorporating clinical, radiological and electroencephalographic (EEG) variables have shown promise in selected cohorts [10]. However, they are seldom implemented in everyday practice, partly due to their complexity, lack of validation or insufficient specificity for the mild-to-moderate TBI population.

This diagnostic gap is particularly concerning given the current absence of evidence-based prophylactic therapies for PTE. Consequently, early identification of individuals at high risk is paramount for implementing timely surveillance, optimizing resource allocation and facilitating inclusion in future trials targeting epileptogenesis [11,12]. Furthermore, the increasing availability of machine learning and real-time decision-support platforms opens new avenues for transforming predictive insights into actionable clinical pathways.

In this review, we aim to comprehensively synthesize the available literature on the incidence, risk factors and predictive models of PTE in patients with mild and moderate TBI. By systematically analyzing findings from population-based cohorts, imaging studies and neurophysiological investigations, we seek to (1) elucidate the true burden of PTE in these under-characterized subgroups and (2) propose a clinically applicable, structured decision-support tool to assist clinicians involved in TBI and epilepsy care, including neurologists, neurosurgeons, emergency physicians, traumatologists, rehabilitation specialists and primary care providers. The aim is not limited to characterizing early seizures but rather to enabling comprehensive risk stratification for post-traumatic epilepsy in patients with mild and moderate TBI.

## 2. Materials and Methods

This study represents a structured narrative review and evidence synthesis, conducted with the aim of assessing the incidence and predictors of post-traumatic epilepsy (PTE) following mild and moderate brain injury (TBI), as well as to develop a clinically applicable decision-support tool. The methodological approach adhered to established best practices for narrative evidence synthesis in clinical research, ensuring both transparency and reproducibility in data collection and analysis.

A comprehensive literature search was carried out across multiple electronic databases, including PubMed, MEDLINE and Google Scholar, covering studies published between 1985 and 2024. The research strategy employed a combination of controlled vocabulary and free-text terms, including but not limited to: “post traumatic epilepsy”, “mild traumatic brain injury”, “moderate traumatic brain injury”, “seizure risk”, “epilepsy prediction”, “contusion”, “intracranial hemorrhage” and “early seizures”. In addition to the primary search, backward citation tracking and manual hand searches were used to identify additional relevant articles that may not have been captured by electronic databases.

Eligible studies were selected based on predefined inclusion and exclusion criteria. Studies were considered for inclusion if they were original, peer-reviewed investigation reporting on either the incidence of risk factors of epilepsy following mild and moderate TBI and if they clearly defined injury severity using Glasgow Coma Scale (GCS) or neuroimaging criteria. Both adult and pediatric populations were considered, provided that quantitative data on PTE risk or predictive modeling were available. Studies were excluded if they focused exclusively on severe TBI (GCS ≤ 8) or penetrating head trauma, if they were case reports, abstracts from scientific meetings, non-English publications or if they lacked sufficient follow-up data or seizure outcome measures.

Although our review followed predefined inclusion and exclusion criteria, the decision to conduct a narrative rather than a systematic review inevitably introduces the potential for selection bias. We attempted to mitigate this by prioritizing high-quality cohort and registry studies and by cross-validating extracted data across multiple sources. Nevertheless, the heterogeneity of available study and the narrative synthesis approach may limit the comprehensiveness of our literature capture.

To ensure consistency across data extraction, TBI severity was classified using harmonized criteria derived from established definitions in the literature. Mild TBI (mTBI) was defined as injury resulting in a GCS score between 13 and 15, with or without transient loss of consciousness or post-traumatic amnesia and encompassing both uncomplicated cases and those with abnormal neuroimaging findings, commonly referred to as “complicated mTBI”. Moderate TBI was defined as injury resulting in a GCS score between 9 and 12, or alternatively, as GCS ≥ 13 in the presence of significant imaging abnormalities such as subdural hematoma (SDH), contusions, subarachnoid hemorrhage (SAH) or radiological evidence of midline shift.

We did not include “previous seizure history” as an independent predictor, because most studies excluded patients with prior epilepsy diagnoses. We acknowledge this omission as a methodological limitation, as prior seizure history may influence the likelihood of PTE in real-world populations.

For each included study, a structured data extraction protocol was applied. Extracted variables included sample size, demographic characteristics, injury classification, PTE incidence rates stratified by severity, duration of follow-up, identified independent risk factors (such as early seizures, hemorrhage, psychiatric history and contusion burden), as well as EEG findings and neuroimaging predictors when available. Studies reporting predictive models or nomograms were further evaluated for the presence of multivariable adjustment, model calibration and external validation, with specific attention to tools such as those developed by Wang et al. [11]. All extracted data were compiled into tabular format and subjected to descriptive synthesis to facilitate comparative analysis across cohorts.

Based on the aggregated evidence, a rule-based clinical decision algorithm was developed to stratify the risk of PTE following mild or moderate TBI. The algorithm integrated key predictors across five domains: injury severity, early seizure occurrence, neuroimaging findings (with emphasis on contusion location and volume, as well as hematoma type), clinical and demographic risk factors (e.g., male sex, psychiatric comorbidities, duration of unconsciousness) and EEG abnormalities. Each variable was assigned a weight proportional to its reported odds ratio or hazard ratio across multiple studies. The resulting algorithm was implemented using the Stream lit platform to facilitate interactive, real-time application in clinical environments.

Although the study does not constitute a systematic review with formal meta-analysis, methodological rigor was ensured by prioritizing high-quality cohort studies, population-based registries and peer-reviewed predictive models. Where available, associations were extracted from multivariable-adjusted analyses to minimize confounding. Studies with ambiguous definitions of TBI or incomplete seizure outcome data were acknowledged in the narrative but were not used in the algorithm’s risk weighting.

Several clinical and imaging factors have been identified as risk factors for PTE following TBO. These include early post-traumatic seizures, contusions, subdural hematomas, skull fractures, prolonged loss of consciousness or post-traumatic amnesia and older age. The selection of these factors is based on their recurrence and reported significance across multiple studies.

Finally, as this investigation is based exclusively on secondary analysis of data from previously published, publicly available sources, no ethical approval was required.

## 3. Results

The initial database search identified a broad pool of studies. Titles and abstracts were screened to exclude investigations focusing on severe or penetrating TBI, studies lacking sufficient follow-up and those without stratified seizure outcomes. Additionally, articles were excluded at full-text review due to methodological limitations or incomplete data reporting. Ultimately, 24 studies met the predefined inclusion criteria and formed the basis for incidence estimates and risk factor analysis, encompassing over 100,000 patients. While precise screening numbers were not systematically documented at each stage, this process followed a structured and transparent approach consistent with narrative review methodology.

A summary of the characteristics of the 24 included studies is presented in Table S1 in the Supplementary Materials, including patient demographics, TBI severity, incidence rates and relevant clinical predictors.

### 3.1. Incidence of PTE Following Mild and Moderate TBI

The incidence of PTE following mild and moderate TBI varies considerably across studies, influenced by study design, population characteristics, follow-up duration and injury definitions. In one of the largest cohort studies to date, Wang et al. reported PTE rates of 1.7% among individuals with mTBI and 2.3% among those with moderate TBI, over an average follow-up of 5.6 years [11]. Similarly, Zhao et al. identified epilepsy in 3.6% of mTBI cases and 6.9% in moderate TBI within three years of injury [13]. Burke et al. found a 0.9% incidence among patients with a GCS of 13–15, which increased to 2.1% when hemorrhagic lesions were present on CT imaging and reached 5.3% in patients with moderate TBI [3].

In pediatric cohorts, Petridis et al. observed that children diagnosed with concussion had a 6.0% incidence or early PTE, rising to 10.6% in those who experienced moderate loss of consciousness and 12.0% in cases complicated by SDH [14]. Laaksonen et al. reported a striking contrast between children requiring neurosurgical intervention, who had a 12.0% incidence of epilepsy, and those with non-operated mild injuries, where the incidence remained below 1.5% [15].

Findings from large-scale registry studies further corroborate these trends (Table 1). Pyrzowski et al. demonstrated a significantly elevated hazard ratio (HR) of 3.44 for epilepsy following a second mTBI [7]. Puvanachandra et al. reported an incidence of 2.6% in uncomplicated mTBI and as high as 12.9% in focal brain injuries [16]. Synthesizing international data, Verellen et al. estimated the cumulative long-term risk of epilepsy at 2.1% for mTBI, 4.2% for moderate TBI and 16.7% for severe cases [4]. However, not all studies found a measurable risk: Christensen et al. reported no epilepsy cases after 7.6 years of follow-up in a cohort of 330 patients with strictly defined mTBI, suggesting that PTE risk is highly contingent on clinical and radiological criteria [6].

**Table 1:** PTE Incidence by TBI Severity.

Study	Population/Setting	mTBI PTE Incidence	Moderate TBI PTE Incidence
[9]	Malaysia cohort	5.1%	10.4%
[13]	China cohort	4.4%	7.6%
[6]	Sweden national cohort	<1% (normal imaging)	N/A
[17]	China pediatric cohort	1.4% (overall)	2.6% (higher contusion load)
[18]	Population review (multi-country)	1.9–4.4%	4.2–7.6%

### 3.2. Key Risk Factors for PTE in Mild and Moderate TBI

Among the most consistent predictors of PTE, early post-traumatic seizures, defined as those occurring within the first 7 days following injury, emerged as a dominant factor. Several studies, including those by Wang et al. and Tubi et al., reported odds ratios ranging from 8 to 50 for this variable [11,17]. Ngadimon et al. also highlighted early seizures, along with intraparenchymal hemorrhage, as independent and robust predictors of later epilepsy [9].

Neuroimaging findings significantly influenced risk estimates across cohorts. SDH, intracerebral hemorrhage (ICH) and especially contusions in the temporal lobe were consistently associated with high risk of PTE. Garner et al. and Ritter et al. underscored lesion burden and anatomical distribution as key contributors to epileptogenesis [3,19]. Tubi et al. found that having three or more contusions or a midline shift greater than 5 mm significantly elevated the probability of developing epilepsy [17].

Demographic and clinical variables also play a role. Male sex, advanced age, loss of consciousness lasting more than 30 min, psychiatric illness, prior history of TBI and the need for neurosurgical intervention were repeatedly identified as risk-enhancing factors [4,8,11]. Laaksonen et al. and Christensen et al. emphasize the impact of pre-existing psychiatric comorbidities, including depression and anxiety, on seizure development [6,15]. Table 2 outlines key demographic and clinical predictors of PTE identified across studies.

EEG abnormalities, particularly early epileptiform discharges or focal slowing, were identified as early biomarkers of epileptogenesis in several studies. Pyrzowski et al. and Arndt et al. showed that EEG performed within the first five days post-injury could provide valuable prognostic information [7,12]. Additionally, hospital-acquired infections such as pneumonia were identified as independent risk factors in moderate-to-severe TBI cases, as demonstrated by Chen et al. [20].

Advanced imaging studies have begun to delineate specific lesion topographies predictive of epileptogenesis. Akrami et al. combined MRI with machine learning approaches and identified bilateral temporal and right occipital lesions as high-risk patterns [21]. In parallel, predictive modelling has evolved substantially. Wang et al. developed a validated nomogram that achieved a concordance index (C-index) of 0.897 for predicting PTE [11], while Tubi et al. proposed a logistic regression model with an area under the curve (AUC) of 0.97 [17]. Khalili et al. suggested using the Glasgow Outcome Scale-Extended (GOS-E) as a screening tool for PTE risk, citing high negative predictive value [22]. Table 3 provides an overview of validated predictive models for PTE used in different populations.

### 3.3. Development of the Clinical Decision Tool

Recognizing the heterogeneity of PTE risk in patients with mild and moderate TBI, one of the principal objectives of this study was to translate available evidence into a pragmatic clinical decision-support tool. The goal was to allow individualized risk stratification and guide clinicians in determining the need for close surveillance, early EEG assessment or potential inclusion in antiepileptogenic trials.

The tool was constructed based on triangulated data from 24 high-quality peer-reviewed studies encompassing over 100,000 patients. Five core domains emerged constantly as strong contributors to PTE risk: injury severity, early seizures, neuroimaging features, clinical-demographic risk factors and EEG abnormalities.

Injury severity was categorized as mild (GCS 13–15) and moderate (GCS 9–12 or GCS ≥ 13 accompanied by significant imaging abnormalities such as hemorrhage, contusions or midline shift). Notably, moderate TBI was associated with PTE incidence rates exceeding 10% in some cohorts [3,9].

Early post-traumatic seizures were found to be among the most powerful predictors of PTE. Reported odds ratios ranged from 8 to over 50 [11,17,19], and this domain received the highest weight in the scoring framework. Similarly, neuroimaging findings such as SDH, EDH, SAH, ICH, frontal or temporal contusions, multiple contusions (≥3), midline shift greater than 5 mm and depressed skull fractures were incorporated as high-risk features [3,9,17,19].

Clinical variables further enriched risk stratification. Factors such as prolonged LOC, male sex, age above 65 years, psychiatric history, ICU admission, prior TBI, family history of epilepsy and the need for neurosurgical intervention were all considered [4,8,9,11,13]. Early EEG findings with epileptiform discharges or focal slowing, especially when recorded within the first week post-injury, were also strongly predictive of future seizures [7,21].

Each variable was assigned a relative weight proportional to its reported odds or hazard ratio in the literature. The detailed scoring system, including specific criteria and their corresponding point values, is presented in Table 4.

The assigned OR (Odds Ratio) and HR (Hazard Ratio) values are extracted from peer-reviewed studies and reflect the relative risk of developing PTE following TBI. Higher scores correspond to stronger risk factors, with the combination of brain contusion and subdural hematoma representing the highest risk. LOC/PTA ≥ 24 h early post-traumatic seizures are also consistent clinical predictors. While minor variations exist across studies, the scoring system offers a standardized framework for PTE risk stratification in both clinical and research contexts.

The scoring system was then calibrated to stratify patients into three categories: low risk (score ≤ 2), from whom routine follow-up is sufficient, moderate risk (score 3–5), from whom outpatient neurology consultation and possible EEG are recommended and high risk (score ≥ 6 or any early seizure), from whom immediate EEG validation, specialist input and consideration of antiepileptic drug initiation may be warranted. These risk categories, along with their corresponding total score ranges, are summarized in Table 5.

The risk stratification approach and corresponding management recommendations are summarized in Table 6.

The recommendation for considering AED use in high-risk patients without seizures was derived from evidence demonstrating substantially elevated odds ratios for PTE in the presence of multiple high-risk features, combined with clinical practice reports suggesting potential benefit of early prophylaxis in selected patients. However, this remains a provisional suggestion pending prospective validation.

**Figure 1:**
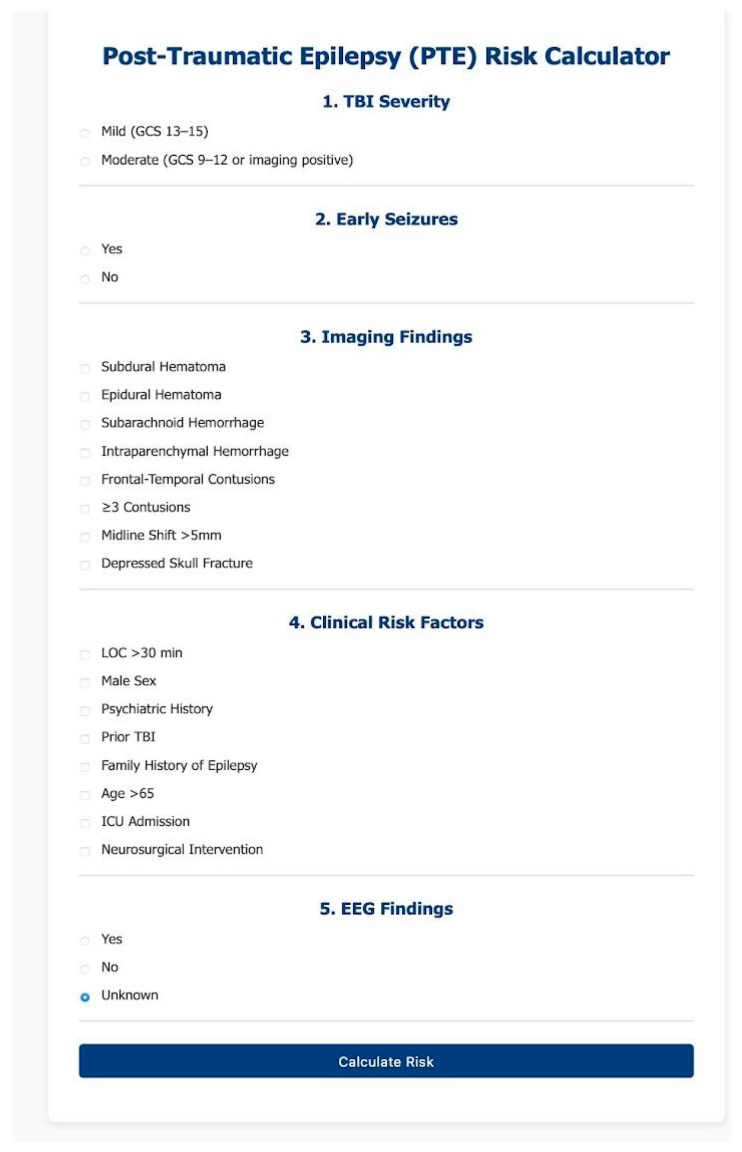
Post-Traumatic Epilepsy (PTE) Risk Calculator—Web Interface.

Importantly, the tool was designed to retain clinical utility even in settings where certain data points (e.g., EEG) are unavailable. This ensures its adaptability across diverse clinical environments, from emergency departments to neurology clinics. Figure 1 presents the interface of the web-based Post-Traumatic Epilepsy Risk Calculator developed for clinical use.

This figure shows the deployed user interface of the Post-Traumatic Epilepsy Risk Calculator, implemented using Streamlit and hosted online. The form includes five structured sections: (1) TBI severity (mild or moderate), (2) presence of early post-traumatic seizures, (3) neuroimaging findings, (4) clinical risk factors, and (5) EEG abnormalities considered included epileptiform discharges (sharp waves, spikes), focal slowing and early periodic discharges identified within the first week post-injury.

Users input relevant clinical data through radio buttons and checkboxes. Upon submission, the tool computes a cumulative risk score and provides a clinical risk category (low, moderate, or high) along with corresponding recommendations for follow-up, EEG referral, or intervention. The design prioritizes clarity, accessibility, and applicability in both clinical and research settings.

## 4. Discussion

PTE remains a serious and underrecognized long-term sequela of TBI, with substantial neurocognitive, psychosocial and economic consequences. While the link between severe TBI and epilepsy is well-established, growing evidence indicates that even mild and moderate TBI can confer a significant epileptogenic risk, particularly when compounded by certain clinical and radiological features [3,4,9,11,17]. This review synthesizes current knowledge from over 100,000 patients across diverse cohorts and contributes to a more nuanced understanding of PTE risk in these underappreciated populations.

### 4.1. Incidence Patterns and Stratification of Risk

The incidence of PTE following mild TBI shows substantial variability depending on the presence or absence of imaging abnormalities. While patients with strictly defined, uncomplicated mTBI exhibit very low rates of epilepsy, often <1% [6], those with early post-traumatic seizures, intracranial hemorrhages or focal contusions face a dramatically increased risk, reaching up to 10% [3,11,13]. In contrast, moderate TBI, particularly as defined by a GCS score of 9–12 or positive neuroimaging, consistently demonstrates PTE rates ranging between 6% and 12% [9,11]. These findings justify the classification of moderate TBI as a high-risk category, deserving of closer monitoring and proactive intervention.

Importantly, early post-traumatic seizures emerged as one of the most powerful predictors of later epilepsy across nearly all datasets reviewed. Studies by Wang et al., Tubi et al. and others consistently report hazard ratios exceeding 4, and in some cases greater than 50 [11,17,19]. Additionally, high-risk imaging features, such as SDH, frontal or temporal contusions, midline shift and the presence of multiple (>3) hemorrhagic lesions were also repeatedly linked with heightened PTE risk [9,17,19]. These findings underscore that structural injury patterns, especially in vulnerable cortical regions, may be as relevant as GCS-based severity in forecasting epileptogenesis.

### 4.2. Clinical Risk Factors and Electrophysiological Correlates

Beyond neuroimaging, several demographic and clinical variables have been consistently associated with increased PTE susceptibility. Male sex, older age, loss of consciousness exceeding 30 min and a prior history of TBI or psychiatric disorders (such as depression or PTSD) all independently correlate with elevated risk [4,8,9,11,13]. These associations likely reflect a complex interplay between systemic vulnerability, neural plasticity and stress-related neuropathology.

EEG findings, although not universally applied in all studies, offer valuable predictive information. Abnormal early EEG patterns, particularly epileptiform discharges and focal slowing within the first week post-injury, were shown to correlate with later seizure onset [7,21]. However, the limited accessibility and heterogeneity in EEG use across clinical settings remain significant barriers to routine integration into prognostic workflows.

### 4.3. Translation into Clinical Practice: Development of a Decision Tool

A central innovation of this study lies in the development of a rule-based, evidence-informed clinical decision tool designed to operationalize seizure risk stratification in real-world settings. By integrating clinical, radiological and electrophysiological variables reported across the literature, the tool enables clinicians to assign patients to low-, moderate- and high-risk categories with minimal data requirements.

Unlike complex nomograms or machine learning classifiers that may be difficult to interpret or implement at the bedside (e.g., Akrami et al., Wang et al.), our tool was specifically designed for usability, transparency and applicability in emergency, rehabilitation and outpatient neurology settings. It provides actionable guidance for further evaluation, such as EEG referral, specialist follow-up or prophylactic antiepileptic therapy consideration, particularly in patients flagged as high risk.

Compared with existing predictive models, such as the validated nomogram by Wang et al. and the pediatric logistic regression model proposed by Tubi et al., our decision tool emphasizes simplicity and bedside applicability. More sophisticated approaches, including Akrami et al.’s MRI-based machine learning classifiers and Ritter et al.’s multivariate prognosis models, demonstrate high accuracy but are limited by the need for advanced diagnostics or complex statistical computation [18,20]. Our framework therefore complements, rather than replaces, these tools by offering a pragmatic option suitable for routine clinical environments while awaiting further validation.

In terms of integration into clinical workflows, the tools could be deployed at key decision points, such as emergency department discharge, inpatient neurology rounds or rehabilitation follow-up visits. Implementation would likely require minimal training as the input variables are routinely collected in standard TBI assessment. However, successful adoption will depend on embedding the algorithm into electronic health record systems or decision-support platforms, ensuring that it augments rather than disrupts existing clinical pathways [22,23].

### 4.4. Limitations and Considerations

Several limitations must be acknowledged. First, despite the comprehensiveness of our literature synthesis, heterogeneity in study methodologies, inclusion criteria and follow-up durations introduces variability in the reported incidence and risk estimates. Second, advanced neurodiagnostic markers, such as volumetric MRI or quantitative EEG, were not consistently available across all cohorts, potentially underestimating their utility in prediction. Third, although our decision tool is based on robust associations and reported effect sizes, it has yet to undergo prospective validation.

While the proposed decision tool provides a structured framework for early risk stratification, it should not yet be considered ready for clinical implementation. Prospective validation in diverse populations is required to confirm predictive accuracy and generalizability. Moreover, training requirements and integration into existing workflows must be carefully evaluated before adoption in clinical practice. Until such evidence is available, the tool should be regarded as a hypothesis-generating instrument rather than a prescriptive guideline.

### 4.5. Implications and Future Directions

Future research should focus on validating this risk model prospectively in multicenter TBI cohorts, including underrepresented populations such as children, older adults and military veterans. Integration with electronic health record systems may facilitate automated scoring and flagging of high-risk cases. Furthermore, ongoing advancements in neuroimaging (e.g., diffusion tensor imaging, connectivity-based biomarkers) and machine learning could further refine predictive accuracy and open new avenues for individualized antiepileptogenesis strategies [17,24].

The inclusion of psychiatric variables in our model also suggests that interdisciplinary approaches, combining neurology, psychiatry and neurorehabilitation, may be necessary to fully address the multifactorial nature of PTE. Ultimately, early identification and tailored surveillance protocols may mitigate long-term disability and improve outcomes for TBI survivors at risk of epilepsy.

## 5. Conclusions

Post-traumatic epilepsy is a significant long-term complication of mild and moderate TBI, particularly in patients with high-risk features such as early seizures, intracranial hemorrhage or focal contusions. Our review identifies consistent clinical, radiological and electrophysiological predictors and integrates them into a pragmatic decision-making support tool for risk stratification. This tool has the potential to enhance early identification and follow-up planning, but prospective validation is essential before routine clinical implementation.

## Figures and Tables

**Table 2:** Predictors of Post-Traumatic Epilepsy.

Risk Factor	Evidence from Studies
Older Age (>65 years)	[6,13]
Psychiatric History	[13]
Family History of Epilepsy	[6]
Prior TBI	[8]
ICU Admission	[15]

**Table 3:** Predictive Models for PTE.

Model/Tool	Input Variables	AUC/Accuracy	Utility
[17] Pediatric Nomogram	GCS, contusion number, early seizures	AUC 0.97	Pediatric risk tool; useful for stratifying long-term follow-up
[22] GOS-E model	GOS-E score at discharge	Accuracy 89.2%	Quick bedside prediction using discharge score
[11]	Sex, neurosurgical procedure, hypoxic injury	AUC not stated	Nomogram for chronic DOC patients with severe TBI

**Table 4:** Risk Scoring Criteria for Post-Traumatic Epilepsy Tool.

Risk Factor	Criteria	Points Assigned	OR (95% CI)	HR (95% CI)	References
Brain contusion + Subdural hematoma	Both present	12	OR = 2.29 (Ritter et al., 2016)	HR = 2.29 (Ngadimon et al., 2024)	[9,19]
Brain contusion only	Present	5	OR = 1.8–2.1 (Ritter et al., 2016)	HR ≈ 1.65 (Ngadimon et al., 2024)	[9,19]
Subdural hematoma only	Present	6	OR = 2.29 (Ritter et al., 2016); OR = 1.64 (95% 0.96–2.78, Kahili et al., 2021)	HR ≈ 1.58 (Ngadimon et al., 2024)	[9,19,22]
Skull fracture	Linear or depressed (age > 5)	2	OR = 1.88 (95% CI 0.92–3.80, Kahili et al., 2021)	HR ≈ 1.45 (Ngadimon et al., 2024)	[9,22]
LOC or PTA	≥24 h	2	OR = 3.6 (Tubi et al., 2019); OR = 1.5 (95% CI 1.1–2.0, Sødal et al., 2024)	HR = 1.5 (95% CI 1.1–2.0, Sødal et al., 2024)	[8,17]
Age	≥65 years	2	OR = 2.9 (Kahili et al., 2021)	HR ≈ 2.13 (Yu et al., 2021)	[2,22]
Early post-traumatic seizure	Within 1 week of injury	1	OR = 3.6 (Ritter et al., 2016); OR = 3.3 (95% CI 2.3–4.9, Sødal et al., 2024)	HR = 3.3 (95% CI 2.3–4.9, Sødal et al., 2024)	[8,19]

**Table 5:** Risk Categories by Score Range.

Risk Category	Total Score Range
Low Risk	0–2
Moderate Risk	3–6
High Risk	7–12
Very High Risk	≥13

**Table 6:** Risk Stratification.

Risk Category	Criteria	Management Recommendations
Low Risk	mTBI, normal imaging, no early seizures, no risk factors	Discharge with safety education; no further EEG or AEDs
Moderate Risk	Moderate TBI or mTBI + 1–2 risk factors or minor hemorrhage	Follow-up within 3 months; consider early EEG; monitor for delayed seizures
High Risk	Early seizures, multiple contusions, temporal lobe lesion, 3+ risk factors, EEG+	Serial follow-up at 1, 3, 6, 12 months; consider AED trial; patient education

## Data Availability

The original contributions presented in this study are included in the article/supplementary material. Further inquiries can be directed to the corresponding author.

## Supplementary Materials

The following supporting information can be downloaded at: https://www.mdpi.com/article/10.3390/reports8040193/s1, Table S1: Characteristics of the 24 included studies on post-traumatic epilepsy after mild and moderate TBI.

## References

[1] Fordington S., Manford M. (2020). A review of seizures and epilepsy following traumatic brain injury. J. Neurol..

[2] Yu T., Liu X., Sun L., Wu J., Wang Q. (2021). Clinical characteristics of post-traumatic epilepsy and the factors affecting the latency of PTE. BMC Neurol..

[3] Garner R., La Rocca M., Vespa P., Jones N., Monti M.M., Toga A.W., Duncan D. (2019). Imaging biomarkers of posttraumatic epileptogenesis. Epilepsia.

[4] Zhong J., Lan Y., Sun L., Zhao Z., Liu X., Shi J., Ren L., Zuo Q., Wei X., Dou X. (2025). Global prevalence of post-traumatic epilepsy in traumatic brain injury patients: A systematic review and meta-analysis (1997–2024). Neuroscience.

[5] Freire M.A.M., Rocha G.S., Bittencourt L.O., Falcao D., Lima R.R., Cavalcanti J.R.L.P. (2023). Cellular and Molecular Pathophysiology of Traumatic Brain Injury: What Have We Learned So Far?. Biology.

[6] Christensen J., Pedersen M.G., Pedersen C.B., Sidenius P., Olsen J., Vestergaard M. (2009). Long-term risk of epilepsy after traumatic brain injury in children and young adults: A population-based cohort study. Lancet.

[7] Pyrzowski J., Kałas M., Mazurkiewicz-Bełdzińska M., Siemiński M. (2024). EEG biomarkers for the prediction of post-traumatic epilepsy—A systematic review of an emerging field. Seizure.

[8] Sødal H.F., Nordseth T., Rasmussen A.J.O., Rosseland L.A., Stenehjem J.S., Gran J.M., Helseth E., Taubøll E. (2024). Risk of epilepsy after traumatic brain injury: A nationwide Norwegian matched cohort study. Front. Neurol..

[9] Ngadimon I.W., Mohan D., Shaikh M.F., Khoo C.S., Tan H.J., Chamhuri N.S., Cheong W.L., Aledo-Serrano A., Yong L.L., Lee Y.M. (2024). Incidence and predictors of posttraumatic epilepsy and cognitive impairment in patients with traumatic brain injury: A retrospective cohort study in Malaysia. Epilepsia.

[10] Wang X., Zhong J., Lei T., Chen D., Wang H., Zhu L., Chu S., Liu L. (2021). An Artificial Neural Network Prediction Model for Posttraumatic Epilepsy: Retrospective Cohort Study. J. Med. Internet Res..

[11] Wang X.P., Zhong J., Lei T., Wang H.J., Zhu L.N., Chu S., Chen D., Liu L. (2021). Development and external validation of a predictive nomogram model of posttraumatic epilepsy: A retrospective analysis. Seizure.

[12] Arndt D.H., Lerner J.T., Matsumoto J.H., Madikians A., Yudovin S., Valino H., McArthur D.L., Wu J.Y., Leung M., Buxey F. (2013). Subclinical early posttraumatic seizures detected by continuous EEG monitoring in a consecutive pediatric cohort. Epilepsia.

[13] Zhao Y., Wu H., Wang X., Li J., Zhang S. (2012). Clinical epidemiology of posttraumatic epilepsy in a group of Chinese patients. Seizure.

[14] Petridis A.K., Doukas A., Maslehaty H., Mehdorn H.M. (2012). Predictors and incidence of posttraumatic seizures in children and adolescents after brain injury. Clin. Pract..

[15] Laaksonen J., Ponkilainen V., Kuitunen I., Möttönen J., Mattila V.M. (2023). Association between pediatric traumatic brain injury and epilepsy at later ages in Finland: A nationwide register-based cohort study. Epilepsia.

[16] Puvanachandra P., Hyder A.A. (2009). The burden of traumatic brain injury in Asia: A call for research. Lancet Neurol..

[17] Tubi M.A., Lutkenhoff E., Blanco M.B., McArthur D., Villablanca P., Ellingson B., Diaz-Arrastia R., Van Ness P., Real C., Shrestha V. (2019). Early seizures and temporal lobe trauma predict post-traumatic epilepsy: A longitudinal study. Neurobiol. Dis..

[18] Yeh C.C., Chen T.L., Hu C.J., Chiu W.T., Liao C.C. (2013). Risk of epilepsy after traumatic brain injury: A retrospective population-based cohort study. J. Neurol. Neurosurg. Psychiatry.

[19] Ritter A.C., Wagner A.K., Szaflarski J.P., Brooks M.M., Zafonte R.D., Pugh M.J., Fabio A., Hammond F.M., Dreer L.E., Bushnik T. (2016). Prognostic models for predicting posttraumatic seizures during acute hospitalization, and at 1 and 2 years following traumatic brain injury. Epilepsia.

[20] Chen Z., Laing J., Li J., O’Brien T.J., Gabbe B.J., Semple B.D. (2024). Hospital-acquired infections as a risk factor for post-traumatic epilepsy: A registry-based cohort study. Epilepsia Open.

[21] Akrami H., Cui W., Kim P.E., Heck C.N., Irimia A., Jerbi K., Nair D., Leahy R.M., Joshi A.A. (2024). Prediction of Post Traumatic Epilepsy Using MR-Based Imaging Markers. Hum. Brain Mapp..

[22] Khalili H., Kashkooli N.R., Niakan A., Asadi-Pooya A.A. (2021). Risk factors for post-traumatic epilepsy. Seizure.

[23] Mithal A., Sehgal M., Newey C., Ems D., Florio V., Singh G. (2025). Prevalence of Seizures in Hospitalizations with Traumatic Brain Injury: A U.S. Population-Based Study. Neurotrauma Rep..

[24] Karlander M., Ljungqvist J., Zelano J. (2021). Post-traumatic epilepsy in adults: A nationwide register-based study. J. Neurol. Neurosurg. Psychiatry.

