# Supplementary material for: Post-Traumatic Epilepsy After Mild and Moderate Traumatic Brain Injury: A Narrative Review and Development of a Clinical Decision Tool
Source: Reports (MDPI). 2025 Sep 29;8(4):193. doi: 10.3390/reports8040193 (PMC12643447; doi:10.3390/reports8040193)
Supplement: Supplementary file 1 [file reports-08-00193-s001.zip › reports-3846700-supplementary.pdf]

Table S1. Characteristics of the 24 included studies on post-traumatic epilepsy after mild and moderate TBI

Study (author, year)	Population/ Setting	Sample size	Age (mean/range)	TBI severity	Early seizure incidence	PTE incidence	Imaging findings	Psychiatric illness	Family history of epilepsy	Neurosurgical intervention	EEG abnormalities
[1]	Narrative review of seizures/epilepsy after TBI	N/A (review)	N/A	Covers mild–severe; summarizes literature	Varies by severity; review (no single %)	Summarizes literature (e.g., higher with severe TBI)	Summarizes CT/MRI predictors; no primary data	Discussed as comorbidity; no primary data	Not specifically quantified	Discussed (e.g., craniectomy) contextually	Discusses early epileptiform activity in TBI
[2]	Single-center adult PTE cohort, China	251 PTE patients	≥18 years; mean ~ (NR exact)	Mixed severities among PTE cases	NR	Study of PTE cases; no denominator	Lesions (frontal/temporal) associated with shorter latency	NR	NR	Operative treatment associated with shorter latency	NR
[3]	Review of imaging biomarkers of posttraumatic epileptogenesis	N/A (review)	N/A	Covers moderate–severe predominantly	N/A	N/A	Summarizes MRI/DTI/fMRI candidates	Not primary focus	Not primary focus	Not primary focus	Not primary focus
[4]	Systematic review & meta-	Meta-analysis (k studies)	Varies by include	All severities	Meta-estimate	Meta-estimate of global	Aggregated; NR	NR	NR	NR	NR

	analysis, global TBI (1997–2024)		d studies		s (NR here)	prevalen ce					
[5]	Review of cellular/mol ecular TBI pathophysio logy	N/A (review)	N/A	Covers experime ntal & clinical TBI	N/A	N/A	Mechanistic focus, not imaging	Discusse d mechanis tically	N/A	N/A	N/A
[6]	Danish nationwide children & young adults	Large registry cohort (exact NR here)	Childre n & young adults	Mild–severe (coded)	NR	Higher in TBI vs controls; varies by severity	Registry; NR	NR	NR	NR	NR
[7]	Systematic review of EEG biomarkers for PTE prediction	N/A (systemati c review)	Varies	Varies	Reported across included studies	Reported across included studies	Not applicable	NR	NR	NR	Focus: epileptif orm discharg es, HFOs, aperiodi c markers
[8]	Nationwide matched cohort, Norway (2011–2015 index; follow-up to 2017)	Hospitaliz ed TBI cohort; exact NR here	Adults (all ages)	Hospitali zed TBI (implies moderate –severe predomi nance)	NR	Cum. incidenc e 3.1% (2y), 4.0% (5y) in TBI; controls lower	NR	Adjusted for psychiatr ic comorbid ity	NR	NR	NR

[9]	Single-center retrospective cohort, Malaysia (TBI patients)	487 TBI admissions (example scale)	Adults (NR exact)	Mixed; moderate–severe predominance	NR	Reported; NR exact here	Hemorrhage/contusion predictors reported	Cognitive impairment assessed	NR	Craniotomy/craniectomy analyzed	NR
[10]	Single-center retrospective cohort for ANN model (China)	1278 TBI patients (training+test; NR exact split)	Adults (NR exact)	Mixed	NR	Outcome for model (incidence NR)	CT findings incorporated as predictors	NR	NR	Surgery included as variable	NR
[11]	Retrospective cohort to build/validate nomogram (China)	1745 (dev) + 175 ext. validation (example scale)	Adults (NR exact)	Mixed	NR	Model outcome; NR	CT hematoma/contusion included	NR	NR	Included as predictor	NR
[12]	Consecutive pediatric TBI ICU cEEG cohort, single center (UCLA)	87 children	Pediatric	Moderate–severe (ICU)	Early PTS 42.5%; subclinical 16.1%	NR (short-term EEG study)	NR	NR	NR	NR	Electrographic seizures in 16.1%
[13]	Nationwide pediatric TBI vs fracture controls,	71,969 pTBI; 64,856 controls	<18 years	Hospitalized pTBI (mixed)	NR	CIR 0.5% at 2y; 1.5% at 15y (pTBI). Higher	NR (registry)	NR	NR	Operated n=337; 12.0% 15y CIR	NR

	Finland (1998–2018)					with neurosurgery.					
[14]	Pediatric/adolescent brain injury, single center (Germany)	NR	Children & adolescents	Mixed	NR	NR	NR	NR	NR	NR	NR
[15]	Commentary on TBI burden in Asia	N/A (commentary)	N/A	N/A	N/A	N/A	N/A	N/A	N/A	N/A	N/A
[16]	Longitudinal cohort of moderate–severe TBI, UCLA	n≈120 (NR exact)	Adults	Moderate–severe	Early seizures predicted PTE	NR exact; increased with temporal lobe trauma	Temporal lobe traumatic lesions predictive	NR	NR	NR	NR
[17]	Multi-center TBI cohorts for prognostic models (US)	Model cohorts with modest n (NR exact)	Adults	Mixed; acute hospitalized and follow-up	Model for acute PTS: poor discrimination	Year 1 & 2 models: fair–good discrimination	CT findings included as predictors	NR	NR	Cranial surgery noted as important predictor	NR
[18]	Multisite imaging dataset of TBI survivors (US)	n≈180 (NR exact here)	Adults	Mixed	NR	Outcome label for ML; NR exact	MRI lesion volume, rs-fMRI connectivity, ALFF predict PTE	NR	NR	NR	NR

[19]	Swedish nationwide register, adults hospitalized with TBI vs matched controls (2000–2010)	111,947 TBI; 325,881 controls	Adults	Hospitalized TBI (mixed)	NR	10-year risk 4.0% (TBI) vs 0.9% controls	Registry; injury type stratified	Adjusted in models	NR	NR	NR
[20]	US National Inpatient Sample (2016–2020)	Adult TBI hospitalizations; NR exact here	Adults	All hospitalized TBI	Prevalence of concomitant seizures during admission reported	Not addressed (in-hospital seizures)	NR	NR	NR	NR	NR
[21]	Taiwan NHIRD retrospective cohort (TBI vs non-TBI)	Large registry; NR exact here	All ages	Mild, moderate, severe coded groups	NR	Adjusted HRs: skull fracture 10.6; severe 5.05; mild 3.02 vs non-TBI	NR	Sex differences reported; broader comorbidity adjusted	NR	NR	NR
[22]	Single-center Chinese TBI cohort	2,826 TBI; 141 PTE	Adults (NR exact)	Mixed	0.8% PTS during acute phase	5.0% developed PTE	Hemorrhage/contusion patterns reported	NR	NR	NR	NR

[23]	Australian state-wide trauma registry, moderate–severe TBI	Registry cohort (NR exact here)	Adults	Moderate–severe	NR	11% at ~2 years overall; higher with hospital-acquired infection	NR	Adjusted for comorbidities	NR	NR	NR
[24]	Iran, severe non-penetrating civilian TBI	Large single-center cohort (NR exact)	Adults	Severe	NR	Reported; risk factors evaluated	Depressed skull fracture, intracranial hematoma explored	NR	NR	Analyzed	NR

NR = Not reported
